# Mutational profile of ZBTB16‐RARA‐positive acute myeloid leukemia

**DOI:** 10.1002/cam4.3904

**Published:** 2021-05-27

**Authors:** Emiliano Fabiani, Laura Cicconi, Anna Maria Nardozza, Antonio Cristiano, Marianna Rossi, Tiziana Ottone, Giulia Falconi, Mariadomenica Divona, Anna Maria Testi, Ombretta Annibali, Roberto Castelli, Vladimir Lazarevic, Eduardo Rego, Pau Montesinos, Jordi Esteve, Adriano Venditti, Matteo Della Porta, William Arcese, Francesco Lo‐Coco, Maria Teresa Voso

**Affiliations:** ^1^ Department of Biomedicine and Prevention University Tor Vergata Rome Rome Italy; ^2^ UniCamillus‐Saint Camillus International University of Health Sciences Rome Italy; ^3^ Unit of Hematology Santo Spirito Hospital Rome Italy; ^4^ Cancer Center ‐ IRCCS Humanitas Clinical & Research Hospital and Humanitas University Milan Italy; ^5^ Department of Translational and Precision Medicine and Hematology Sapienza University Rome Italy; ^6^ Hematology and Stem Cell Transplantation Unit University Campus Biomedico Rome Italy; ^7^ Department of Biomedical and Clinical Sciences Luigi Sacco Hospital Milan Italy; ^8^ Department of Hematology, Oncology and Radiation Physics Skåne University Hospital Lund Sweden; ^9^ Department of Internal Medicine Medical School of Ribeirao Preto Sau Paulo Brazil; ^10^ Hematology Department Hospital Universitari i Politècnico la Fe Valencia Spain; ^11^ Department of Hematology Hospital Clínic de Barcelona Barcelona Spain

**Keywords:** AML, ARID1A, NGS, ZBTB16‐RARA

## Abstract

**Background:**

The *ZBTB16‐RARA* fusion gene, resulting from the reciprocal translocation between *ZBTB16* on chromosome 11 and *RARA* genes on chromosome 17 [t(11;17)(q23;q21)], is rarely observed in acute myeloid leukemia (AML), and accounts for about 1% of retinoic acid receptor‐α (*RARA)* rearrangements. AML with this rare translocation shows unusual bone marrow (BM) morphology, with intermediate aspects between acute promyelocytic leukemia (APL) and AML with maturation. Patients may have a high incidence of disseminated intravascular coagulation at diagnosis, are poorly responsive to all‐*trans* retinoic acid (ATRA) and arsenic tryoxyde, and are reported to have an overall poor prognosis.

**Aims:**

The mutational profile of *ZBTB16‐RARA* rearranged AML has not been described so far.

**Materials and methods:**

We performed targeted next‐generation sequencing of 24 myeloid genes in BM diagnostic samples from seven *ZBTB16‐RARA*+*AML*, 103 non‐RARA rearranged AML, and 46 APL. The seven *ZBTB16‐RARA*‐positive patients were then screened for additional mutations using whole exome sequencing (*n* = 3) or an extended cancer panel including 409 genes (*n* = 4).

**Results:**

*ZBTB16‐RARA*+AML showed an intermediate number of mutations per patient and involvement of different genes, as compared to APL and other AMLs. In particular, we found a high incidence of *ARID1A* mutations in *ZBTB16‐RARA*+AML (five of seven cases, 71%). Mutations in *ARID2* and *SMARCA4*, other tumor suppressor genes also belonging to SWI/SNF chromatin remodeling complexes, were also identified in one case (14%).

**Discussion and conclusion:**

Our data suggest the association of mutations of the *ARID1A* gene and of the other members of the SWI/SNF chromatin remodeling complexes with *ZBTB16‐RARA*+AMLs, where they may support the peculiar disease phenotype.

## Graphical abstract

This is the first report showing the mutational landscape of the rare ZBTB16‐RARA rearranged acute myeloid leukemia (AML). We identified a high frequency of ARID1A mutations, suggesting the involvement of the SWI/SNF chromatin remodeling complexes in the clinical presentation, acute promyelocytic leukemia‐like morphology and all‐*trans* retinoic acid/arsenic trioxide treatment resistance of ZBTB16‐RARA rearranged AMLs.

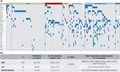

## 1 INTRODUCTION

Acute promyelocytic leukemia (APL) accounts for 5%–8% of all acute myeloid leukemia (AML). The hallmark of APL is the reciprocal balanced *t*(15;17)(q24.1;q21.2) translocation, which fuses the promyelocytic leukemia (*PML*) gene on chromosome 15 and the retinoic acid receptor‐α (*RARA*) on chromosome 17. The resulting oncoprotein, PML‐RARA, is the key player of APL pathogenesis acting as repressor of transcription of multiple RARA target genes, thus leading to a differentiation block, and uncontrolled proliferation of atypical promyelocytes.^1^ For this reason, the definition of APL with *PML‐RARA* has been included in “the 2016 revision of the World Health Organization classification of myeloid neoplasms and acute leukemia,” in order to stress the prominent role of the *PML‐RARA* rearrangement to define this AML subtype.^2^ APL is nowadays the most curable type of AML, with over 90% of patients cured by a chemotherapy‐free regimen, combining all‐*trans* retinoic acid (ATRA) and arsenic trioxide.^1^, ^2^, ^3^, ^4^, ^5^, ^6^, ^7^

The *RARA* gene may fuse to partner genes other than *PML* in less than 1% of AMLs, presenting with clinical and/or morphological features suggestive of APL, and therefore, often referred to as APL‐like AML.^2^, ^3^ Fourteen variant translocations involving the RARA gene have been described: *ZBTB16‐RARA*, *NPM‐RARA*, *NuMA‐RARA*, *STAT5B‐RARA*, *PRKAR1A‐RARA*, *BCOR‐RARA*, *FIP1L1‐RARA*, *OBFC2A‐RARA*, *GTF2I‐RARA*, *IRF2BP2‐RARA*, *FNDC3B‐RARA*, *STAT3‐RARA*, *TBLR1‐RARA*, *and TGF‐RARA*.^8^ Among these, the *ZBTB16‐RARA* fusion, previously known as *PLZF‐RARA*, results from the reciprocal translocation between *ZBTB16* (zinc finger and BTB domain containing 16) on chromosome 11, and the *RARA* gene on chromosome 17 [*t*(11;17)(q23;q21)]. This is the most frequently reported variant, accounting for about 1% of all APL‐like AML.^8^, ^9^ The bone marrow (BM) of patients carriers of this rare translocation shows a high rate of normal or dysplastic promyelocytes, and rare or absent Auer rods. Clinically, patients may have disseminated intravascular coagulation at diagnosis, are poorly responsive to ATRA, and are reported to have an overall poor prognosis.^3^, ^8^, ^10^

Due to its extreme rarity, the mutational profile of *ZBTB16‐RARA* rearranged AML has not been described so far. The aim of our work was to dissect the molecular landscape of *ZBTB16‐RARA* AML, as compared to controls including a) classical APLs with *PML‐RARA* rearrangement, and b) other AMLs, in order to define biological differences sustaining the disease phenotype, and eventually identify “druggable” mutations.

## 2 MATERIALS AND METHODS

### 2.1 Patients' characteristics

In total, 156 AML patients, diagnosed between 2005 and 2019, were included in this study.

Seven cases were characterized by the t(11;17)(q23;q21) translocation resulting in the *ZBTB16‐RARA* rearrangement. These cases were collected from seven institutions in Europe (Italy, Spain, and Sweden), and Brazil, and common case report forms (including laboratory features at diagnosis, type of treatment, and response as well as follow‐up data) were used to obtain and share patient information among all participating centers.

As a comparative group, we studied 46 patients consecutively diagnosed with APL at the Policlinico Tor Vergata, in Rome. Median age was 45 years (range 19–79 years), and there were 19 (41.3%) females and 27 (58.7%) males. At diagnosis, 29 patients (63.0%) were classified as standard‐risk and 17 (37.0%) as high‐risk APL.^11^

Additionally, we included 103 patients consecutively diagnosed with non‐*RARA* rearranged AML, of a median age of 69 years (range 1–88, 41 females and 62 males). Of these, 42 (40.8%) patients had a normal karyotype, 9 (8.7%) had recurrent abnormalities (4 inv(16)(p13.1q22) or t(16;16)(p13.1;q22), 4 inv(3)(q21.3q26.2) or t(3;3)(q21.3;q26.2) or 3q26‐rearrangements, and 1 t(6;9)(p23;q34.1)), 24 (23.3%) had a complex karyotype, 22 (21.4%) had other abnormalities, while karyotype was not available in six AML cases (5.8%).

All patients gave informed consent for the participation in the study, according to the declaration of Helsinki. The study was approved by the ethics committee of participating centers.

### 2.2 Molecular genetic analyses

Bone marrow mononuclear cells (BM‐MNC) were isolated from all patients by Ficoll gradient centrifugation using lympholyte‐H (Cedarlane). Nucleic acids (DNA and RNA) were extracted from BM‐MNC collected at the time of diagnosis. DNA was extracted using the QIAamp DNA Mini Kit (Qiagen) and quantified using a Qubit Fluorometer (Life Technologies), whereas total RNA was extracted using the RNeasy Mini Kit (Qiagen AG). Complementary DNA used for reverse transcription quantitative PCR was synthesized using the QuantiTect Reverse Transcription Kit (Qiagen AG), in accordance with the manufacturer's instructions. Molecular studies to verify the presence of the *ZBTB16‐RARA* fusion transcript were conducted according to a previously published protocol.^10^ The *FLT3* mutational status (ITD and TKD) was evaluated by PCR amplification, followed by capillary electrophoresis using the ABI 3130 Instrument (Thermo Fischer Scientific) as reported.^12^ DNA extracted from nails was available for three *ZBTB16‐RARA*+AML patients (UPN2, UPN6 and UPN7) and was used as germline control.

### 2.3 Next‐generation sequencing assays

The MYeloid Solution panel (SOPHiA GENETICS) was used to screen for somatic mutations samples from seven ZBTB16‐RARA+AML and 103 AML. Thirty genes known to be frequently mutated in myeloid malignancies were studied according to the manufacturer's protocol. The resulting captured libraries were further processed on a MiniSeq or MySeq sequencing platform (Illumina). Generated FASTQ sequencing files were then uploaded on the SOPHiA DDM platform for analysis by SOPHiA technology. Only mutations identified by the SOPHiA DDM platform as highly or potentially pathogenic were considered for analysis. The sensitivity of this next‐generation sequencing (NGS) method is about 1%. Mutational profiling of BM samples collected from 46 APL patients was performed using the TruSeq Custom Amplicon and sequenced on the Illumina platform following the manufacturer's instructions (Illumina).^13^ The FASTQ files were further processed using the Sequence Pilot software version 4.1.1 (JSI Medical Systems) for alignment and variant calling.

The functional significance of the somatic mutations was checked on the public Catalogue Of Somatic Mutations In Cancer (COSMIC) v69 database. Functional interpretation was performed using SIFT 1.03, PolyPhen 2.0 and MutationTaster 1.0 algorithms. Single‐nucleotide polymorphisms (SNPs), annotated according to the National Center for Biotechnology Information Single Nucleotide Polymorphism Database (NCBI dbSNP) were deleted from the analysis. The detection limit for variants was 3% variant allele frequency (VAF).

The seven *ZBTB16‐RARA*‐positive patients were then screened for additional mutations using whole exome sequencing (*n* = 3 patients) or the Ion AmpliSeq™ Comprehensive Cancer Panel (Thermo Fisher Scientific) (*n* = 4 patients with low amount of available DNA). This panel studies the exons of 409 tumor suppressors and oncogenes (complete gene list at http://assets.thermofisher.com/TFS‐Assets/CSD/Reference‐Materials/ion‐ampliseq‐cancer‐panel‐gene‐list.pdf; ThermoFisher), using ultra‐high multiplex PCR combined with targeted sequencing library construction. According to the manufacture's protocol, Switch solution and DNA Ligase were used to bind purified DNA target amplicons to Dual Barcode Adapters. Libraries were then prepared for template preparation on the Ion OneTouch™ System and sequencing on the Ion PGM™ Sequencer (Thermo Fisher Scientific). Variant annotations were performed using the Torrent Suit Software and the Ion Reporter Software (Thermo Fisher Scientific). Only mutations identified as highly or potentially pathogenic were considered.

**FIGURE 1:**
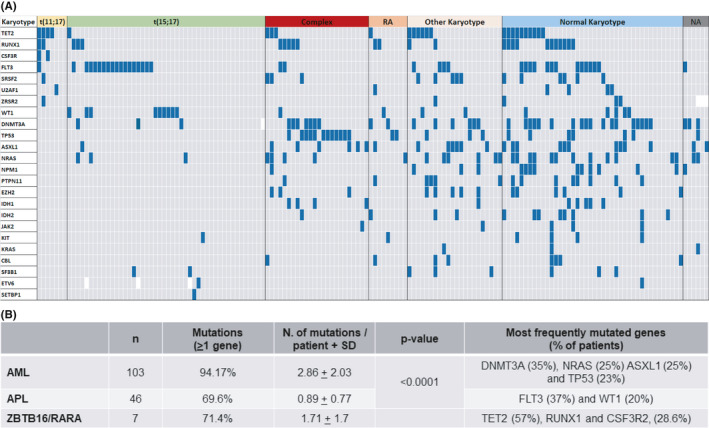
Mutational profiles of *ZBTB16‐RARA*+, as compared to APL and to other AML subtypes. (A) Mutational profile of AML patients using targeted NGS including 24 myeloid‐specific genes. Data from seven *ZBTB16‐RARA*+is shown and compared to that of 46 APL and 103 non‐RARA rearranged AML, grouped according to their karyotype. Complex karyotype: 24 (three or more abnormalities); RA: 4 inv(16)(p13.1q22) or t(16;16)(p13.1;q22), 4 inv(3)(q21.3q26.2) or t(3;3)(q21.3;q26.2) or 3q26‐rearrangements, and 1 t(6;9)(p23;q34.1). Other karyotype: 22; Normal karyotype: 42; karyotype not available (NA): 6. (B) Comparison in the number of mutations (mean ± SD; *p*‐value) and the most frequently mutated genes in the three AML subtypes. AML, acute myeloid leukemia; APL, acute promyelocytic leukemia; NGS, next‐generation sequencing; RA, recurrent abnormalities (2); SD, standard deviation

Whole exome sequencing was performed using the SureSelectXT Human All Exon Kit (Agilent Technologies) and sequenced on the Illumina platform following the manufacturer's instructions (Illumina). The FASTQ files were further processed according to GATK best practices for alignment and variant calling. The identified variants were functionally annotated using ANNOVAR. We excluded from analysis variants in non‐coding regions, synonymous variants, variants present in highly repetitive regions or variants annotated to be present in ExAC, and 1000 Genomes of European origin with minor allele frequency > 0.01. The limit for variant detection was 5% VAF. *ARID1A* mutations identified at a VAF greater than 15% in *ZBTB16‐RARA* AML were validated by Sanger sequencing using specifically designed primers, reported in Table S1, and the ABI 3130 instrument (Thermo Fischer Scientific). A detailed flowchart of molecular tests performed on *ZBTB16‐RARA*+AML, non‐RARA rearranged AML and APL is reported in supplementary (Figure 1S).

### 2.4 Statistical analysis

D'Agostino and Pearson omnibus normality test, Shapiro–Wilk normality test, and Kolmogorov–Smirnov test were used to evaluate non‐Gaussian distributions.

In case of parametric distribution, the associations between the prevalence of mutations, and patient characteristics were studied using *t*‐test and Fisher's exact test (when frequencies were lower than 5).

Non‐parametric tests (Kruskal–Wallis and Mann–Whitney) were performed in case of non‐Gaussian distribution. All cut‐offs for testing the null hypothesis were set at *p* ≤ 0.05.

All tests were performed using the GraphPad Prism 6 software (GraphPad Software Inc Corporation).

The statistical analysis for each NGS test has been describe in the above paragraph. To assess pathogenic variants, the following software were used: SIFT 1.03, PolyPhen 2.0, MutationTaster 1.0 and ANNOVAR.

Polymorphisms were excluded according to NCBI dbSNP, COSMIC v69 database, ExAC, and 1000 Genomes of European origin.

## 3 RESULTS

### 3.1 Patient characteristics and mutational profiling of *ZBTB16‐RARA*+ AMLs

We collected samples from seven patients with a *ZBTB16‐RARA*+ AML (three females and four males, median age: 75 years, range 14–83 years). Two cases (28%) were classified as therapy‐related AML due to previous exposure to radiochemotherapy for solid tumors. In particular, UPN 3 underwent radiotherapy for a previous breast cancer, while UPN 7 had a history of rheumatoid arthritis treated with hydroxychloroquine and methotrexate. Detailed characteristics of patients with *ZBTB16‐RARA*+ AML are reported in Table 1. Karyotype analysis showed the presence of t(11;17)(q23;q21) as the sole chromosomal abnormality in 5 cases, while UPN 7 had an additional del(5)(q13;q31), while karyotype was not available in one case. The presence of ZBTB16‐RARA fusion transcript was confirmed in all cases by the central laboratory at Tor Vergata University by RT‐PCR, using previously published methods.^10^ Flow cytometry characterization was available in six of these cases and showed strong positivity for CD33 and CD13, together with CD34 and HLA‐DR negativity in all cases. All five patients with available data were found to express CD56, as compared to two of 20 patients with classical APL (Mann–Whitney *p* = 0.0004).

**TABLE 1:** Characteristics of patients with ZBTB16‐RARA+ AML

UPN	Age	Sex	Immuno‐phenotype	Morphology	WBC (10^9^/L)	Plts (10^9^/L)	Fibrinogen (mg/dl)	Karyotype	Genetics	Induction therapy	Consolidation therapy	Relapse	Outcome (months after diagnosis)
1	7	M	CD33+, CD117+, CD13+, CD56+, MPO+, CD34‐, HLA−DR−	M3	20.0	138.0	85.0	46, XY, *t*(11;17) (q23;q21) [18]	*ZBTB16‐RARA*	ATRA +ICE	ATRA +GO	None	Alive, CR (86)
2	83	M	CD45+, CD33+, CD117+, CD13+, CD4+, CD56+, MPO+, CD34−, HLA−DR−	M3	6.0	34.0	338.0	46, XY, *t*(11;17) (q23;q21) [20]	*ZBTB16‐RARA*	ATRA +HU	ATRA +L‐DAC	None	Alive, PR (12)
3	77	F	—	M3	8.0	—	—	46, XX, *t*(11;17) (q23;q21) [18]	*ZBTB16‐RARA*	ATRA +DNR + ARA‐C	DNR +ARA‐C (2 cycles)	Relapsed at 36 m	Alive, CR (36)
4	75	F	CD33+, CD117+, CD13+, CD34−, HLA−DR−	M3	44.0	64.0	154.0	46, XX, *t*(11;17) (q23;q21) [12]	*ZBTB16‐RARA*	ATRA	—	—	Dead (1)
5	38	M	CD45+, CD33+, CD117+, CD13+, CD56+, CD34−, HLA−DR−	M3	10.1	65.0	344.0	—	*ZBTB16‐RARA*	ATRA +DNR	1st H‐DAC +DNR; 2nd H‐DAC	None	Alive, CR (9)
6	76	M	CD33+, CD117+, CD13+, CD56+, MPO+, CD34−, HLA−DR−	M3	44.0	140.0	171.0	46, XY, *t*(11;17) (q23;q21) [15]	*ZBTB16‐RARA*	ATRA +LD ARA‐c	L‐DAC +ATRA	—	Dead, PR (12)
7	50	F	CD33+, CD117+, CD13+, CD56+, CD34−, HLA−DR−	M3	2.9	26.0	312.0	46, XX, del(5)(q13q31), t(11;17)(q23;q 1) [20], 46, XX [10]	*ZBTB16‐RARA*	ATRA +ICE	ATRA +MTZ + ARA‐C (2 cycles)	None	Alive, CR (36)

Abbreviations: ARA‐C, cytarabine; ATRA, all‐trans retinoic acid; CR, complete remission; DNR, daunorubicin; GO, gemtuzumab ozogamycin; H‐DAC, high‐dose cytarabine; HU, hydroxyurea; ICE, idarubicin, cytarabine, etoposide, L‐DAC, low‐dose cytarabine; MTZ, mitoxantrone outcome; PR, partial remission.

All patients received ATRA as part of their treatment, combined with conventional chemotherapy (CHT) approaches in four cases, while two patients received low‐dose cytarabine or hydroxyurea, while single‐agent ATRA was used in one case (Table 1). The four patients treated with conventional CHT/ATRA combinations were alive in CR at a median of 36 months from diagnosis (range 9–86 months).

Using targeted NGS and studying 24 myeloid‐specific genes (listed in Table S2), we found that five of seven *ZBTB16‐RARA*+AML were mutated in at least one gene (71.42%), and the total number of mutated genes was 12 (mean of 1.71 ± SD 1.70 mutations/patient, Figure 1). Median VAF of the mutations was 32.76% (range 1.0%–91.3%). Most frequently mutated genes were: *TET2* (4/7 patients, 57.14%), *RUNX1*, and *CSF3R* (2/7 patients each, 28.57%), while *SRSF2*, *U2AF1*, and *ZRSR2* were mutated in one patient (Figure 1A). Multiple mutations in the *TET2* gene were found in all mutated patients and the mean VAF was 42.8% (range 1.0%–91.3%). Of note, a FLT3‐TKD mutation was present in only one of the *ZBTB16‐RARA*+AML, while none was FLT3‐ITD mutated (data confirmed by PCR amplification, followed by capillary electrophoresis) (Figure 1B).

We then compared the mutational profile of the *ZBTB16‐RARA*+AML to that of BM‐MNC isolated at initial diagnosis in 46 patients with APL and 103 patients with other AML subtypes. In the cohort of 103 AML, we found at least one mutation in 97 patients (94.17%), with a mean of 2.86 mutations/patient (SD: ±2.03). Most frequently mutated genes in AML included *DNMT3A* (36/103 patients, 34.95%), *NRAS* and *ASXL1* (25/103 patients, 24.27%), *TP53* (24/103 patients, 23.30%), *FLT3*, *TET2*, and *RUNX1* (20/103 patients, 19.42%), and *NPM1* (15/103 patients, 14.56%).

APL samples were characterized by a significantly lower number of mutations, with 69.56% of patients with a least one mutation (32/46), and a total of 41 mutated genes, corresponding to meanly 0.89 mutations/patient (SD: ±0.77, Mann–Whitney test, *p* < 0.0001). Only two genes, *FLT3* (17/46, 36.96%) and *WT1* (9/46 patients, 19.57%), were frequently mutated in APL. *FLT3* mutation subtype was ITD in 32.61% and TKD in 8.70% of patients (15 and four of 46 patients, respectively). Two patients (4.35%) were mutated for both ITD and TKD.

### 3.2 Extended mutational landscape of *ZBTB16‐RARA*+AML

**FIGURE 2:**
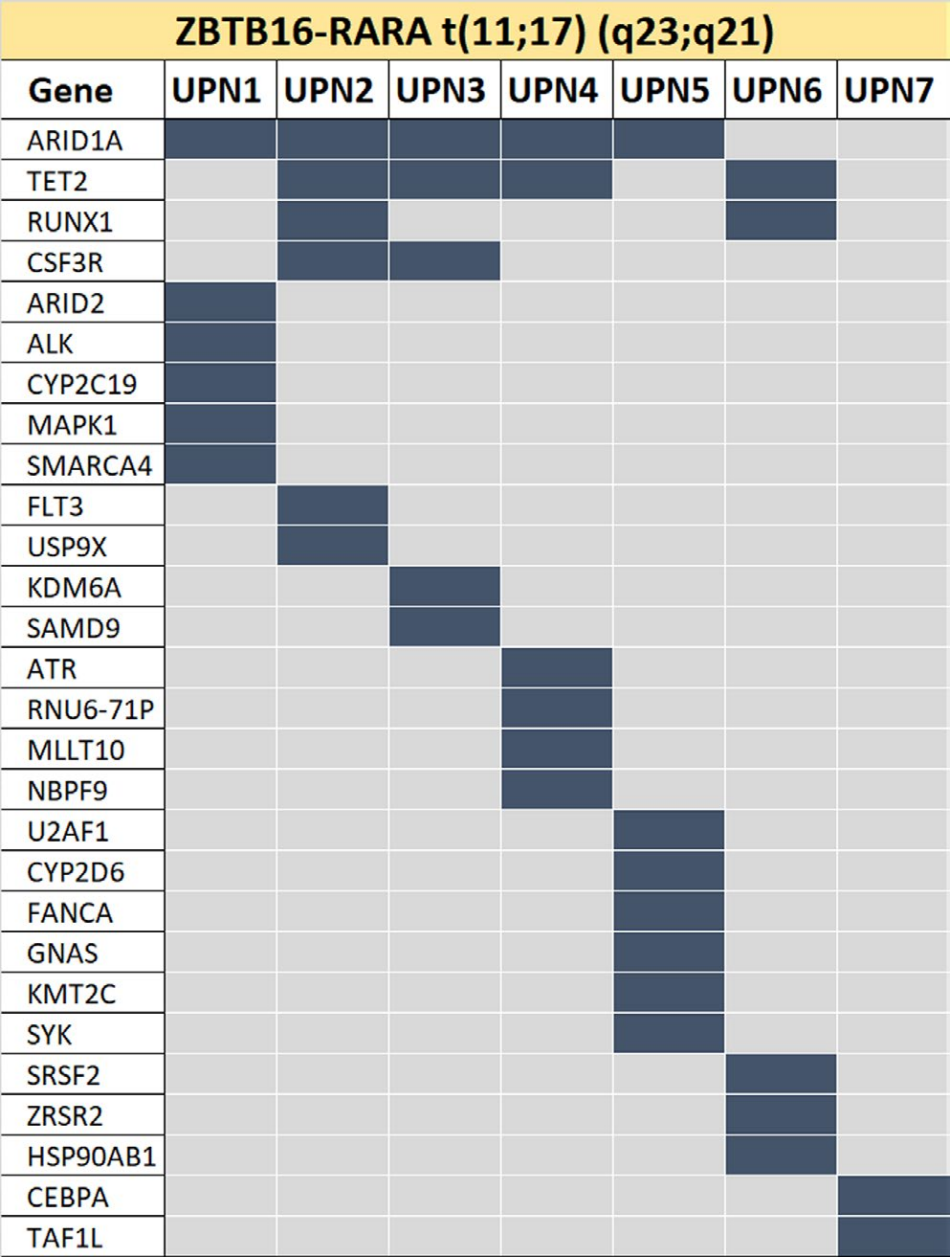
Extended mutational landscape of *ZBTB16‐RARA*+AML. Studying 417 genes, we identified 37 mutations, with a mean of 5.29 (SD ±1.60) mutations per patient. The most frequently mutated genes were *ARID1A* (71%), *TET2* (57%), *RUNX1*, and *CSF3R* (28% each)

We then performed an extended mutational profiling of the seven *ZBTB16‐RARA*+AML, to try to identify alternative mutations involved in the disease phenotype or as potential therapy targets. We identified 37 mutations in 417 genes (Figure 2; Table S3). The mean number of mutations was 5.29, SD ±1.60 per patient and at least two mutated genes were identified per patient (median 6, range 2–7). The most frequently mutated gene was *ARID1A* (5/7 patients, 71%), followed by *TET2* (4/7 patients, 57%), *RUNX1* and *CSF3R* (2/7 patients, 28%), confirming data derived from the targeted myeloid panel.

**FIGURE 3:**
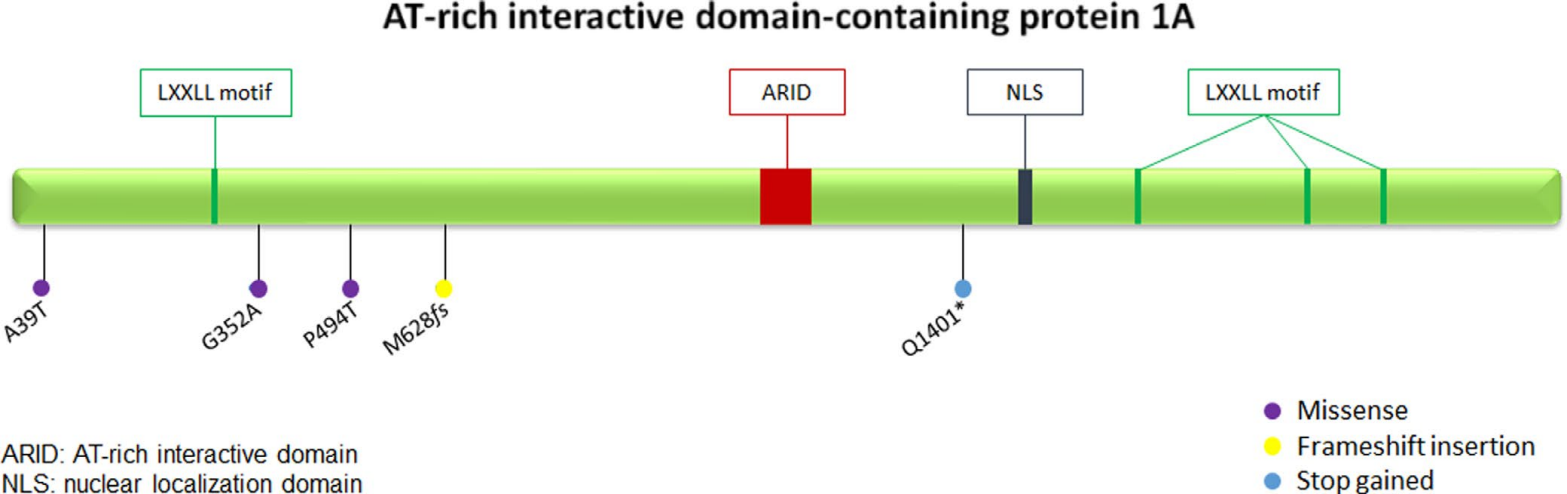
Schematic representation of ARID1A protein domains. Domain information was derived from UniProt database (https://www.uniprot.org/). Location and type of somatic mutations are reported. ARID, AT‐rich interactive domain; LXXLL, leucine‐rich steroid receptor binding motif; NLS, nuclear localization domain

*ARID1A* mutations included four single nucleotide variants (SNV) and one frameshift mutations (Table 2; Figure 3). SNV were *ARID1A*‐Q1401* (VAF 34%) generating a stop codon, *ARID1A*‐G352A (VAF 21%), *ARID1A*‐P494T (VAF 16%), and *ARID1A*‐A39T (VAF 6%), whereas the frameshift mutation was *ARID1A*‐M628*fs* (VAF 31%). Mutation hotspots were not identifiable in the *ARID1A* gene and the mutations did not fall into specific functional domains. The VAF of all identified mutations (median 21%, range 6%–34%) suggests their somatic origin. This was confirmed in UPN2 in whom the germline control resulted negative for *ARID1A*‐Q1401*. Of note, one of these AMLs (UPN1) presented a mutation in three genes belonging to SWI/SNF chromatin remodeling complexes, including *ARID1A*‐M628*fs* (VAF 31%), *ARID2*‐S975P (VAF 45%), and *SMARCA4*‐R1633W (VAF 9%).

**TABLE 2:** Type of ARID1A mutations identified in ZBTB16‐RARA+AML. In “RefSeq Gene” column, the nucleotide variations from reference genome according to their localizations are reported. In “Mutation” column, the nucleotide variations and localizations according to coding regions are reported. In “Amino acid change” column, amino acids changes are reported according to their localizations in coding regions

UPN	*ARID1A* status	RefSeq gene	Mutation	Amino acid change	Variant effect	VAF
1	Mutated	n.5488G>A	c.115G>A	p. Ala39 Thr	Missense	6%
2	Mutated	n.83398C>T	c.4201C>T	p. Gln1401*	Stop gained	34%
3	Mutated	n.6428G>C	c.1055G>C	p. Gly352Ala	Missense	21%
4	Mutated	n.40521C>A	c.1480C>A	p. Pro494 Thr	Missense	16%
5	Mutated	n.41723_41724insAGGG	c.1881_1882insAGGG	p. Met628 fs	Frameshift insertion	31%
6	Wild‐type	—	—	—	—	—
7	Wild‐type	—	—	—	—	—

Abbreviations: RefSeq, reference sequence;VAF, variant allele frequency.

## 4 DISCUSSION

To the best of our knowledge, this is the first report on the molecular landscape of the rare *ZBTB16‐RARA*+AML. Our NGS studies show that the mutational profile of these AMLs is intermediate between APL and other AML subtypes. Differences were evident in the rate of mutations, with *ZBTB16‐RARA*+ cases characterized by about two mutations/patient, while there were a median of 0.9 and 2.9 mutations/patient in samples collected from APL and non‐RARA rearranged AMLs.

Mutational profiles were also different. Indeed, *FLT3* (ITD) and *WT1* mutations were frequent in APL, while other more “typical” AML genes were mutated at very low frequency, in line with results from previous studies,^13^, ^14^, ^15^, ^16^ confirming the specific mutation profile of APL.

The profile of the *ZBTB16‐RARA*+ *AML was* “intermediate” between APL and other AMLs, including recurrent mutations in AMLs, such as those in *TET2*, *RUNX1* and *CSF3R*, and mutations rarely reported in APL, such as *ARID1A*.^15^ Mutations in *ARID1A*, belonging to the SWI/SNF chromatin remodeling complexes, have been previously described in hematological malignancies, including chronic lymphocytic leukemia and hairy cell leukemia, and in solid cancers.^15^, ^16^, ^17^, ^18^, ^19^, ^20^ The highly conserved SWI/SNF multi‐subunit protein complexes, include an ATP‐dependent catalytic subunit that utilizes the energy from ATP hydrolysis to remodel chromatin structure, allowing the access to DNA of transcriptional activators and repressors. As previously shown, *ARID1A* loss may affect expression or stability of other SWI/SNF subunits, resulting in transcriptional dysfunction, disruption of nucleosome sliding activity, and altered recruitment of coactivators and/or corepressors.^21^ Mutations in *ARID1A* and *ARID1B*, which are not included in typical myeloid panels for NGS analysis, may play a functional role and could be responsible for ATRA resistance in *ZBTB16‐RARA*+*AML*. Indeed, silencing of *ARID1A* and *ARID1B* in the APL cell line NB4 impaired differentiation in response to ATRA treatment, suggesting the pivotal role of these genes in the differentiation process toward the granulocytic lineage.^15^, ^22^ The crucial role of *ARID1A* during hematopoietic differentiation has also been demonstrated in mice models, where knockdown of *ARID1A* caused granulocytic differentiation defects in the BM, and the accumulation of immature granulocytes and monocytes in the spleen.^22^ Madan et al. reported a low incidence of mutations in *ARID1A* in APL (5%) and even lower in AML (1%).^15^ In our cohort of *ZBTB16‐RARA*+AMLs, the incidence of *ARID1A* mutations was surprisingly high (71%), which leads us to speculate that the characteristic disease phenotype may be due to these mutations. Indeed, in *ZBTB16‐RARA*+AML, it has previously shown that ATRA degrades the abnormal fusion protein, but does not induce response, demonstrating that the oncoprotein loss is not sufficient to induce differentiation or clinical response.^23^, ^24^, ^25^, ^26^ Similar to Madan et al., as shown in Figure 3, we did not identify mutational hotspots in specific *ARID1A* functional domains, likely pointing out the involvement of the whole coding sequence of this gene in the tight interactions with the other members of the SWI/SNF chromatin remodeling complexes.^15^

Of note, in addition to *ARID1A*, in one patient we also found mutations in *ARID2* and *SMARCA4* genes, which also belong to the SWI/SNF chromatin remodeling complexes, suggesting that impairment of this pathway may be a general mechanism of transformation in *ZBTB16‐RARA*+ AML. In this line, SNV or deletions in the AT‐rich interactive domain 2 (*ARID2*) were detected at low frequencies in various tumors,^27^, ^28^, ^29^ and *SMARCA4* (*BRG1*), which is a core component of both SWI/SNF‐A and B complexes, was reported as essential for maintenance of stemness of AML.^30^, ^31^ *SMARCA4* mutations were also identified in patients with rhabdoid tumor predisposition syndrome, characterized by a high risk of developing rhabdoid tumors.^32^ In this line, targeted screening of genes belonging to the SWI/SNF chromatin remodeling complexes may be useful to best asses their role in the pathogenesis and treatment resistance of AML with other rare variant translocations involving the *RARA* gene.

Reduced ARID1A mRNA expression has been associated with poor prognosis in several cancers,^33^ and with decreased intracellular glutathione (GSH) level,^34^ which in turn increases production of reactive oxygen species and DNA damage. Low GSH levels have been shown to increase sensitivity to GSH inhibitors, such as APR‐246, in ARID1A‐deficient ovarian and gastric cancer cell lines.^34^, ^35^ The high prevalence of ARID1A mutations in *ZBTB16‐RARA*+AML may extend the applicability of APR‐246 treatment, which also targets TP53‐mutations, and has shown efficacy in TP53 mutated cancers,^36^, ^37^ including MDS and AML.^38^, ^39^

In conclusion, this is the first report describing that *ZBTB16‐RARA*+AML are characterized by high incidence of *ARID1A* mutations, and suggesting the involvement of the SWI/SNF chromatin remodeling complexes in the clinical presentation of this AML subtype. Further studies will be needed to explore the potential role of APR‐246, likely in combination with other agents, as targeted treatment in ARID1A‐mutated AML patients.

## CONFLICT OF INTEREST

The authors declare no conflict of interest.

## AUTHOR CONTRIBUTION

Maria Teresa Voso, Emiliano Fabiani and Francesco Lo‐Coco made substantial contributions to conception and design of the study; Maria Teresa Voso and Emiliano Fabiani wrote the paper and critically revised the data; Emiliano Fabiani, Laura Cicconi, Anna Maria Nardozza, Antonio Cristiano, Marianna Rossi, Tiziana Ottone, Giulia Falconi and Mariadomenica Divona performed experiments and critically analyzed the data; Anna Maria Testi, Ombretta Annibali, Roberto Castelli, Vladimir Lazarevic, Eduardo Rego, Pau Montesinos, Jordi Esteve, Adriano Venditti, Matteo Della Porta and William Arcese provided samples and clinical data of patients; all authors critically revised and approved the manuscript.

## Supporting information

Supplementary Material

## Data Availability

The data that support the findings of this study will be available upon request to corresponding author.
