# Supplementary material for: Mutational profile of ZBTB16‐RARA‐positive acute myeloid leukemia
Source: Cancer Med. 2021 May 27;10(12):3839–47. doi: 10.1002/cam4.3904 (PMC8209618; doi:10.1002/cam4.3904)
Supplement: Supplementary file 1 — Supplementary Material [file CAM4-10-3839-s001.docx]

**Supplementary Table S1**

Sequence of the primers used for validation of ARID1A mutations identified at a VAF greater than 15% in ZBTB16-RARA AML patients.

**Supplementary Table S2**

List of 24 genes known to be frequently mutated in myeloid neoplasms used for the molecular screening of 156 AML patients

| ASXL1 | CBL | CSF3R | DNMT3A | ETV6 | EZH2 | FLT3 | IDH1 |
| --- | --- | --- | --- | --- | --- | --- | --- |
| IDH2 | JAK2 | KIT | KRAS | NPM1 | NRAS | PTPN11 | RUNX1 |
| SETBP1 | SF3B1 | SRSF2 | TET2 | TP53 | U2AF1 | WT1 | ZRSR2 |

| UPN | Sequence | Position | Accession number | Amplicon |
| --- | --- | --- | --- | --- |
| 2 | Forward: 5’- GTG GGC TTT ATG TCC CTG AGT GCA GAG T-3’  Reverse: 5’- AGT AGC AGC TGT GGC AGT GGC AGG ATA-3’ | 78255-78542 | NG_029965 | 278bp |
| 3 | Forward: 5’-TCC ACC AAC AAC ATG GCG GAC AAC-3’  Reverse: 5’-ACA CAA AGG CTG AAG GAG GGA AGG AG-3’ | 5879-6706 | NG_029965 | 827bp |
| 4 | Forward: 5’-AAA AAC CCA GCT TGG CCG TCC TTC-3’  Reverse: 5’-AGC AAA GTT CTC ATG ACA CTA ACC CCC-3’ | 39932-40652 | NG_029965 | 720bp |
| 5 | Forward: 5’-GGC CAG ACA TTA GCA TTT AAC CCC AAG-3’  Reverse: 5’-GGC AGG GAG ACA GAA CAG ACA TCT AC-3’ | 41387-41932 | NG_029965 | 545bp |

**Supplementary Table S3**

List of 417 genes included in the extended mutational profiling of ZBTB16/RARA rearranged AML.

| *ABL1* | *BAI3* | *CASC5* | *CMPK1* | *EGFR* | *EZH2* | *FOXP4* | *IGF2* | *KEAP1* |
| --- | --- | --- | --- | --- | --- | --- | --- | --- |
| *ABL2* | *BAP1* | *CBL* | *COL1A1* | *EML4* | *FAM123B* | *FZR1* | *IGF2R* | *KIT* |
| *ACVR2A* | *BCL10* | *CCND1* | *CRBN* | *EP300* | *FANCA* | *G6PD* | *IKBKB* | *KLF6* |
| *ADAMTS20* | *BCL11A* | *CCND2* | *CREB1* | *EP400* | *FANCC* | *GATA1* | *IKBKE* | *KRAS* |
| *AFF1* | *BCL11B* | *CCNE1* | *CREBBP* | *EPHA3* | *FANCD2* | *GATA2* | *IKZF1* | *LAMP1* |
| *AFF3* | *BCL2* | *CD79A* | *CRKL* | *EPHA7* | *FANCF* | *GATA3* | *IL2* | *LCK* |
| *AKAP9* | *BCL2L1* | *CD79B* | *CRTC1* | *EPHB1* | *FANCG* | *GDNF* | *IL21R* | *LIFR* |
| *AKT1* | *BCL2L2* | *CDC73* | *CSF1R* | *EPHB4* | *FAS* | *GNA11* | *IL6ST* | *LPHN3* |
| *AKT2* | *BCL3* | *CDH1* | *CSF3R* | *EPHB6* | *FBXW7* | *GNAQ* | *IL7R* | *LPP* |
| *AKT3* | *BCL6* | *CDH11* | *CSMD3* | *ERBB2* | *FGFR1* | *GNAS* | *ING4* | *LRP1B* |
| *ALK* | *BCL9* | *CDH2* | *CTNNA1* | *ERBB3* | *FGFR2* | *GPR124* | *IRF4* | *LTF* |
| *APC* | *BCR* | *CDH20* | *CTNNB1* | *ERBB4* | *FGFR3* | *GRM8* | *IRS2* | *LTK* |
| *AR* | *BIRC2* | *CDH5* | *CYLD* | *ERCC1* | *FGFR4* | *GUCY1A2* | *ITGA10* | *MAF* |
| *ARID1A* | *BIRC3* | *CDK12* | *CYP2C19* | *ERCC2* | *FH* | *HCAR1* | *ITGA9* | *MAFB* |
| *ARID2* | *BIRC5* | *CDK4* | *CYP2D6* | *ERCC3* | *FLCN* | *HIF1A* | *ITGB2* | *MAGEA1* |
| *ARNT* | *BLM* | *CDK6* | *DAXX* | *ERCC4* | *FLI1* | *HLF* | *ITGB3* | *MAGI1* |
| *ASXL1* | *BLNK* | *CDK8* | *DCC* | *ERCC5* | *FLT1* | *HNF1A* | *JAK1* | *MALT1* |
| *ATF1* | *BMPR1A* | *CDKN2A* | *DDB2* | *ERG* | *FLT3* | *HOOK3* | *JAK2* | *MAML2* |
| *ATM* | *BRAF* | *CDKN2B* | *DDIT3* | *ESR1* | *FLT3-TKD* | *HRAS* | *JAK3* | *MAP2K1* |
| *ATR* | *BRD3* | *CDKN2C* | *DDR2* | *ETS1* | *FLT4* | *HSP90AA1* | *JUN* | *MAP2K2* |
| *ATRX* | *BRIP1* | *CEBPA* | *DEK* | *ETV1* | *FN1* | *HSP90AB1* | *KAT6A* | *MAP2K4* |
| *AURKA* | *BTK* | *CHEK1* | *DICER1* | *ETV4* | *FOXL2* | *ICK* | *KAT6B* | *MAP3K7* |
| *AURKB* | *BUB1B* | *CHEK2* | *DNMT3A* | *ETV6* | *FOXO1* | *IDH1* | *KDM5C* | *MAPK1* |
| *AURKC* | *CALR* | *CIC* | *DPYD* | *EXT1* | *FOXO3* | *IDH2* | *KDM6A* | *MAPK8* |
| *AXL* | *CARD11* | *CKS1B* | *DST* | *EXT2* | *FOXP1* | *IGF1R* | *KDR* | *MARK1* |

| *MARK4* | *MYC* | *NTRK3* | *PIK3R1* | *RAF1* | *SF3B1* | *TCF3* | *TSHR* |
| --- | --- | --- | --- | --- | --- | --- | --- |
| *MBD1* | *MYCL1* | *NUMA1* | *PIK3R2* | *RALGDS* | *SGK1* | *TCF7L1* | *U2AF1* |
| *MCL1* | *MYCN* | *NUP214* | *PIM1* | *RARA* | *SH2D1A* | *TCF7L2* | *UBR5* |
| *MDM2* | *MYD88* | *NUP98* | *PKHD1* | *RB1* | *SMAD2* | *TCL1A* | *UGT1A1* |
| *MDM4* | *MYH11* | *PAK3* | *PLAG1* | *RECQL4* | *SMAD4* | *TET1* | *USP9X* |
| *MEN1* | *MYH9* | *PALB2* | *PLCG1* | *REL* | *SMARCA4* | *TET2* | *VHL* |
| *MET* | *NBN* | *PARP1* | *PLEKHG5* | *RET* | *SMARCB1* | *TFE3* | *WAS* |
| *MITF* | *NCOA1* | *PAX3* | *PML* | *RHOH* | *SMO* | *TGFBR2* | *WHSC1* |
| *MLH1* | *NCOA2* | *PAX5* | *PMS1* | *RNASEL* | *SMUG1* | *TGM7* | *WRN* |
| *MLL* | *NCOA4* | *PAX7* | *PMS2* | *RNF2* | *SOCS1* | *THBS1* | *WT1* |
| *MLL2* | *NF1* | *PAX8* | *POT1* | *RNF213* | *SOX11* | *TIMP3* | *XPA* |
| *MLL3* | *NF2* | *PBRM1* | *POU5F1* | *ROS1* | *SOX2* | *TLR4* | *XPC* |
| *MLLT10* | *NFE2L2* | *PBX1* | *PPARG* | *RPS6KA2* | *SRC* | *TLX1* | *XPO1* |
| *MMP2* | *NFKB1* | *PDE4DIP* | *PPP2R1A* | *RRM1* | *SRSF2* | *TNFAIP3* | *XRCC2* |
| *MN1* | *NFKB2* | *PDGFB* | *PRDM1* | *RUNX1* | *SSX1* | *TNFRSF14* | *ZNF384* |
| *MPL* | *NIN* | *PDGFRA* | *PRKAR1A* | *RUNX1T1* | *STK11* | *TNK2* | *ZNF521* |
| *MRE11A* | *NKX2-1* | *PDGFRB* | *PRKDC* | *SAMD9* | *STK36* | *TOP1* | *ZRSR2* |
| *MSH2* | *NLRP1* | *PER1* | *PSIP1* | *SBDS* | *SUFU* | *TP53* |  |
| *MSH6* | *NOTCH1* | *PGAP3* | *PTCH1* | *SDHA* | *SYK* | *TPR* |  |
| *MTOR* | *NOTCH2* | *PHOX2B* | *PTEN* | *SDHB* | *SYNE1* | *TRIM24* |  |
| *MTR* | *NOTCH4* | *PIK3C2B* | *PTGS2* | *SDHC* | *TAF1* | *TRIM33* |  |
| *MTRR* | *NPM1* | *PIK3CA* | *PTPN11* | *SDHD* | *TAF1L* | *TRIP11* |  |
| *MUC1* | *NRAS* | *PIK3CB* | *PTPRD* | *SEPT* | *TAL1* | *TRRAP* |  |
| *MUTYH* | *NSD1* | *PIK3CD* | *PTPRT* | *SETBP1* | *TBX22* | *TSC1* |  |
| *MYB* | *NTRK1* | *PIK3CG* | *RAD50* | *SETD2* | *TCF12* | *TSC2* |  |

**Supplemetary Figure S1**

Flow chart reporting the molecular NGS tests performed in the different group of patients.

**
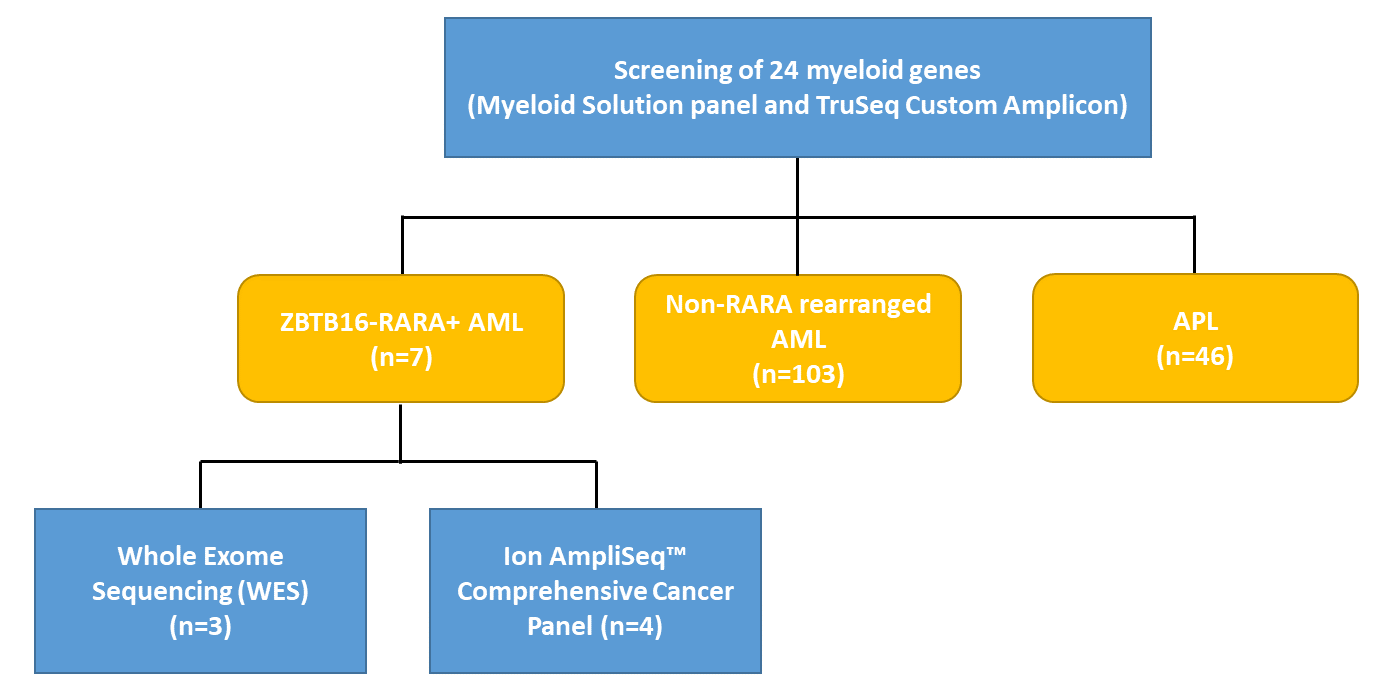
**
